# The Relationship Between Alarm Fatigue and Medical Error Tendency in Intensive Care Unit Nurses: The Mediating Affect of Role Overload

**DOI:** 10.1111/nicc.70121

**Published:** 2025-07-24

**Authors:** Esra Sarioğlu, Mustafa Amarat

**Affiliations:** ^1^ Ordu University Ordu Türkiye

**Keywords:** alarm fatigue, ICU, mediating role, medical error, role overload

## Abstract

**Background:**

Investigating all the reasons that may create a tendency for medical errors in interdisciplinary work due to critical patient care and monitoring in intensive care units is important for reliable health service delivery.

**Aim:**

The primary purpose of this study was to determine the alarm fatigue levels of intensive care nurses, examine the effect of this fatigue on the tendency to make medical errors and determine the mediating role of role overload in the relationship between the two concepts.

**Study Design:**

This cross‐sectional survey study included 250 intensive care nurses from Ordu. In addition to the personal information form, the ‘Nurses Alarm Fatigue Scale’, ‘Nurses Medical Error Tendency Scale’ and ‘Role overload Scale’ were used in the study. After verifying the data's validity and reliability, the SPSS and AMOS package programs were used for the analyses.

**Results:**

The increase in the level of alarm fatigue caused an increase in role overload by 33% and a 9% effect on medical error tendency. The increase in role overload caused a 9% increase in nurses' tendency to medical errors. In addition, it was revealed that their total effect on each other was 11%. There was a positive correlation between role overload, alarm fatigue and medical error tendency, and this relationship was statistically significant.

**Conclusion:**

This research will help us develop effective strategies by better understanding the factors that may lead to medical error tendencies among intensive care nurses responsible for the care of critically ill patients. Although it is not easy to solve alarm fatigue because the alarm response that is not acted upon or the alarm response is impaired is caused by technical, clinician and organisational factors, studies have attempted to be analytical.

**Relevance to Clinical Practice:**

Considering alert fatigue and role overload in reducing the propensity of critical care nurses to make medical errors can significantly increase the accuracy and integrity of patient care and improve outcomes.

**Impact Statements**

- What is known about the topic
  ○ Intensive care nurses' tendency to commit medical errors can cause serious harm to patients and healthcare systems. Low staffing, high role overload, long working hours and stress can lead to medical errors.
  ○ Alarm fatigue is a technological hazard that occurs when healthcare professionals become insensitive to critical warnings due to the frequency of false or unnecessary alarms.
- What this paper adds
  ○ This study is the first to reveal the mediating role between role overload, alarm fatigue and the tendency to make medical errors.
  ○ Based on the research findings, it was concluded that an increase in the level of alarm fatigue in nurses causes an increase in role overload, and an increase in role overload causes an increase in the tendency to make medical errors.
  ○ This study emphasises the importance of role overload and alarm fatigue on the tendency to make medical errors by intensive care nurses.

## 1 Introduction

The ageing population in both Turkey and globally [1] has led to an increased demand for healthcare services, underscoring the need for enhanced capacity and quality in critical care areas such as intensive care units (ICUs). According to the Ministry of Health data, the number of intensive care beds per 10 000 people increased from 4.8 to 5.7 between 2019 and 2022, and the ratio increased from 16.6% to 18.3%. However, compared to OECD countries, although Turkey has a high bed capacity, the number of healthcare personnel remains insufficient [2, 3]. This situation increases the physical, emotional and motivational burden of nurses working in ICUs and puts patient safety and quality of care at risk [4, 5]. ICUs are special units managed by multidisciplinary teams that require advanced technology and 24‐h uninterrupted care [6]. Nurses working in these units are responsible for quickly detecting and intervening in changes in the clinical condition of patients. Therefore, nurses' performance and working conditions directly impact patient safety and treatment outcomes [7]. However, intense workloads, long working hours and stress pave the way for medical errors. In the literature, it has been shown that 53.1% of nurses experience medical errors; the most frequent errors occur in medication administration, and the main reasons for these errors are workload (87.9%) and fatigue (75.9%) [8]. Studies conducted in Turkey also emphasise that insufficient nurses, lack of education and stress trigger medical errors [9, 10]. With technological developments, the number of medical devices used in ICUs has increased, and different alarm systems for each device have caused ‘alarm fatigue’ in nurses. Alarm fatigue is the technological hazard that occurs when healthcare professionals become insensitive to critical warnings due to the frequency of false or unnecessary alarms [11]. Studies have indicated that 80%–99% of alarms are clinically insignificant, which in turn delays nurses' response times [12]. For example, preventable patient deaths due to alarm fatigue in the United States have forced health authorities (ECRI, JCAHO) to develop protocols on this issue. In Turkey, the lack of alarm management training, inadequate noise control and lack of protocols carry the problem to an organisational dimension [13, 14].

ICU nurses experience mental and physical exhaustion due to additional responsibilities such as administrative duties, material management and technological adaptation in addition to patient care. The literature states that 46% of nurses experience performance loss due to noise and 42% due to interruptions such as phone calls from families [15]. Increased role overload reduces nurses' time at the patient's bedside and increases the risk of hospital infections and complications [16]. The imbalance of the nurse–patient ratio in Turkey also multiplies this burden.

In the light of this information, the study's primary purpose was to determine the alarm fatigue levels of nurses working in ICUs, to examine the tendency of this fatigue to medical errors and to determine the mediating role of role overload in the relationship between the two concepts.

## 2 Methods

### 2.1 Study Design

In this study, we acted according to the STROBE guidelines. This was a cross‐sectional study involving surveys of nurses in participating hospitals conducted between May 2024 and June 2024.

### 2.2 Setting and Sampling

The study setting consisted of intensive care nurses (*N* = 359 as of 2024) working at different levels in different institutions in the Ordu province. During the research period, the numbers varied due to reports, maternity leaves and relocations, and these numbers were removed from the setting due to the closure of ICUs. Six surveys were applied to 256 nurses who accepted the research and actively used the convenience sampling method. These were not included in the analyses because they were incomplete and gave incorrect answers to the control questions. Therefore, 250 nurses constituted the sample of the study.

### 2.3 Variables and Measures

The survey technique was used to collect data. In the first stage, the participants answered questions prepared in line with the literature on personal demographic information (gender, age, marital status, education level, etc.), professional characteristics (work type, working hours, working hours in intensive care), characteristics of the ICU they work in (number of intensive care beds, intensive care level, number of patients they are responsible for, which medical device gives the most alarm). Secondly, three scales in the study were used to collect data.

#### 2.3.1 Nurses Alarm Fatigue Questionnaire (NAFQ)

It was developed by Camellia Torabizadeh and her colleagues in 2017 and consists of 13 questions. The validity and reliability of the scale in Turkey was carried out by Kahraman in 2020 with a thesis study, and it was reduced to 9 statements to ensure reliability [17]. An increase in the scale average indicates an increase in the level of alarm fatigue in nurses. A decrease in the score indicates a decrease in the level of alarm fatigue. Cronbach's alpha value was found to be 0.91 on the original scale. It was recorded as 0.80 in Turkish validity.

#### 2.3.2 Medical Error Tendency in Nursing (METN)

The scale developed by Özata and Altunkan in 2009 consists of 5 sub‐dimensions and 49 items for routine record notes of officials in patient care [18]. The low score obtained from the scale is 49, and the high score is 245. The internal results of the scale are pretty high (Cronbach's alpha 0.95), interpreted as reducing officials' medical error transactions within the scope of the total scores. Within the scope of the research, Cronbach's alpha value was determined as 0.86.

#### 2.3.3 Role Overload (RO)

It was developed by Beehr et al. (1976) and consists of three items. The validity and reliability study of the scale in Turkish was carried out by Akbolat et al. (2017) [19]. High scores on the scale mean higher role overload, and low scores mean lower role overload. Cronbach's alpha value was found to be 0.76 on the original scale. It was recorded as 0.75 in the Turkish validity. Within the scope of the research, Cronbach's alpha value was determined to be 0.80.

### 2.4 Statistical Analyses

For statistical analysis, SPSS was used to test the research hypotheses and AMOS. Before moving on to the analyses of the study, confirmatory factor analysis and Cronbach alpha test were performed for all scales used in the study. While all scales met the fit indices (χ^2^/df, TLI, CFI, GFI, AGFI and RMSEA) within the scope of the study, Cronbach alpha values were above 0.80.

Then, the socio‐demographic characteristics of the participants were defined using frequencies and percentages. After this stage, the Pearson correlation coefficient was used to determine the correlations. SPSS Hayes model 4 was used for 95% confidence intervals to test the direct and indirect effects.

### 2.5 Validity and Reliability of Scales

The study used confirmatory factor analysis to test whether the goodness of fit was appropriate. As in the original NAFQ, the single‐factor structure was confirmed in this study. DFA findings, χ^2^/df = 2.921, TLI = 0.915, CFI = 0.948, GFI = 0.997, AGFI = 0.889, RMSEA = 0.080. METN scale, the five‐factor structure was confirmed in this study. DFA findings, χ^2^/df = 1.962, TLI = 0.932, CFI = 0.901, GFI = 0.851, AGFI = 0.898, RMSEA = 0.062. RO scale, the single‐factor structure, was confirmed in this study. DFA findings, χ^2^/df = 0.855, TLI = 0.993, CFI = 0.951, GFI = 0.997, AGFI = 0.983, RMSEA = 0.001. According to these values, it was decided that the goodness of fit of the scales used in the study was appropriate.

## 3 Results

### 3.1 Participant Characteristics

The age range of the participants included in the analysis was 18–54 years (mean = 34.72, SD = 5.01). 20.4% were male and 79.6% were female; most participants (78%) were married. More than 90% of the participants had a bachelor's degree. Table 1 presents the participants' specific demographic data. The proportion of those working in the ICU for 5 years or more was 62.4%.

**TABLE 1:** Distribution of findings regarding demographic and occupational characteristics of ICU.

Variables	Frequency (*n*)	Percentage (%)
Sex		
Female	199	79.6
Male	51	20.4
Age (years)		
18–25	12	4.8
26–30	76	30.4
31–40	83	33.2
≥ 41	79	31.6
Marital status		
Married	195	78
Single	55	22
Education level		
Junior college or below	19	7.6
Bachelor's	204	81.6
Master's or above	27	10.8
Work experience (years)		
0–5	43	17.2
6–10	74	29.6
11–15	50	20.0
≥ 16	83	33.2
Work experience ICU (years)		
0–5	94	37.6
6–10	71	28.4
11–15	49	19.6
≥ 16	36	14.4

### 3.2 Descriptive Statistics and Correlation Analysis

Table 2 shows the relationship between the scales by looking at the Pearson correlation coefficients (*p < 0.05, **p < 0.01). It was determined that the coefficient of relationship between role overload and alarm fatigue in nurses was 0.309 and this relationship was statistically significant at the **p < 0.01 significance level. This result shows a positive and statistically significant relationship between role overload and alarm fatigue in nurses. Alarm fatigue in nurses tends to increase as role overload increases. When the relationship between role overload and medical error tendency in nurses was examined, it was found that the coefficient was 0.144, and this relationship was statistically significant at the 95% confidence level. This result shows a statistically significant relationship between role overload and medical error tendency in nurses. As role overload increases, the tendency to make medical errors in nurses also increases. However, this relationship is weak due to the low correlation coefficient. The correlation coefficient between the following variables, alarm fatigue in nurses and medical error tendency in nurses, is 0.168, and this relationship is statistically significant at the 95% confidence level. This result shows a statistically significant relationship between alarm fatigue and medical error tendency, but this relationship is relatively weak.

**TABLE 2:** Descriptive statistics and correlation analysis.

Variables	Mean	SD	RO	NAFQ	METN
RO	3.500	6402	1		
NAFQ	3.885	5962	309**	1	
METN	1.655	3893	144*	168**	1

Abbreviations: METN, medical error tendency in nursing; NAFQ, a nurses alarm fatigue questionnaire; RO, role overload.

* *p* < 0.05.

** *p* < 0.01.

### 3.3 Mediating Effect Analysis

**FIGURE 1:**
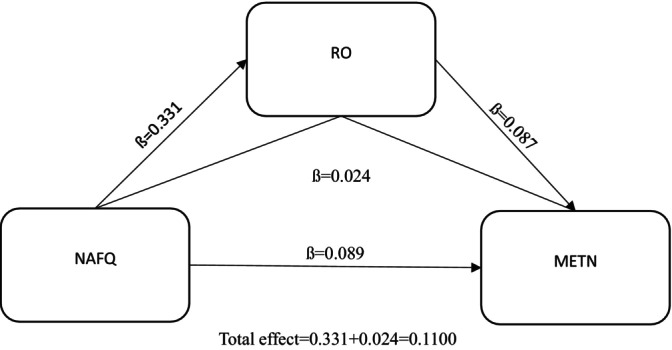
Research model. METN, medical error tendency in nursing; NAFQ, a nurses alarm fatigue questionnaire; RO, role overload.

According to the research results, as seen in Figure 1, the established model was statistically significant. Alarm fatigue has a positive effect on role overload, and the magnitude of this effect is 0.331. In addition, role overload positively affects the tendency to make medical errors (0.087). When other variables are examined according to the model, it is seen that alarm fatigue has a direct positive effect on the tendency to make medical errors at a rate of 0.089. Finally, as seen in the model, the indirect effect of alarm fatigue on the tendency to make medical errors through role overload was determined as 0.110 in total.

## 4 Discussion

Our study found a positive effect on the tendency for medical errors in nurses as alarm fatigue increased. Alarm fatigue in ICU nurses may negatively impact their professional competence. This can make it difficult for nurses to fulfill their responsibilities, reduce their quality of work, and jeopardize patient safety [12, 20]. In one study, it was reported that nurses may experience cognitive overload as a result of alarms, and this may cause medical errors [21]. Another study found that 96% of participants believed alarm sounds negatively affect the quality of patient care and increase the risk of errors. Alarm fatigue may even lead to the overlooking of life‐threatening events [22].

Further, a study showed that ICU nurses responded to 61% of monitor alarms with the involvement of multiple nurses, while 9% did not respond at all, indicating a high alarm burden and low response rate [14]. In addition, another study drew attention to the increase in the clinician's response delay as the number of unactioned alarms increased [23]. In a transplant cardiac ICU, 95%–98% of nurses reported that false alarms were common, disrupted patient care, reduced their trust in alarm systems and often led them to disable alarms inappropriately [24]. Another study conducted in a children's hospital drew attention to the decrease in the time it took for nurses to go to the patient and respond as the number of alarms per shift increased [25].

Our findings also showed that increased alarm fatigue positively affects role overload. Notably, alarm density was associated with a 33% increase in nurse role overload. Frequent false alarms disrupt workflow, require constant attention and force nurses to manage multiple tasks simultaneously, such as attending alarms while delivering care, leading to unfinished tasks, higher infection risks and reduced focus [26]. This elevated role overload may cause delayed alarm responses. Moreover, increased alarm frequency can extend hospital stays, contribute to injuries and lead to patient fatalities [23, 27, 28]. When nurses are engaged in complex caregiving activities or prepare medication, responding to multiple types of alarms can cause cognitive dispersion and increased workload [28]. In another ICU‐based study, noise was reported as the top barrier to performance by 46% of nurses [15].

Our study determined that as the role overload increased, there was a positive effect on the tendency for medical errors in nurses. A study conducted on 1092 nurses with similar impressive results stated that 22% of the nurses made medical errors that would endanger patient safety during their professional lives, and 4% of them stated that patients were harmed due to medical errors [29]. Ten percent of the nurses who made medical errors stated that they delayed the patient's treatment, and 6% stated that the patient experienced side effects. Twenty‐three percent of all nurses stated that they delayed the patient's treatment due to workload, and 20% stated that they made a medical error regarding performing an application without checking the instruments. Of these nurses, 83% stated that they made medical errors due to fatigue, 82% due to the lack of nurses and 75% due to lack of communication with the doctor and other deficiencies [29]. The study on the causes of medical errors includes negligence of healthcare personnel, defective arrangements in healthcare institutions and excessive workload [30]. Ergonomic and structural arrangements resulting from the work system cause fatigue in the employee and an increase in role overload, and it was concluded that this supports the tendency for medical errors [31]. Although our study found a relatively low overall tendency towards errors, this is consistent with previous findings [30, 32, 33, 34, 35].

A study conducted in the literature examined the relationship between the number of alarms and role overload of 26 nurses in a total of 394 nursing shifts in a children's hospital over 2 months, and more than 40 alarms were experienced within 2 h. It has been reported that if the number of alarms is too high and the workload of nurses increases, the time allocated to the patient decreases, causing patient care to be missed and the risk of potential harm to the patient increases [36]. Such circumstances foster fatigue and inattentiveness, which are major contributors to patient safety incidents [37]. Another study examining nurses' working hours and error rates determined that the error rate increased in nurses working more than 12 h a day and 40 h a week [38]. In another study examining the effect of working in shifts and long hours on patient safety, it was determined that working more than 8 h increased the risk of medical error, and working 12 h or more was twice the risk of working 8 h [39]. According to the findings obtained from our research, it was determined that the increase in the alarm fatigue level of nurses can also affect the tendency for medical errors. Based on our study, the increase in nurses' alarm fatigue was associated with an 11% rise in medical error tendency, with role overload acting as a mediator. Alarm fatigue levels in our study were also higher than in previous studies, and this fatigue significantly impacted role overload, which in turn increased the risk of errors during clinical practice. In summary, alarm fatigue can lead to missed alarms, overlooked critical events and delays in care, ultimately increasing the risk of medical errors and jeopardising patient safety.

### 4.1 Limitations

There are several limitations to this study. First, the study sample consisted of only 250 ICU nurses, so the sample size was limited to only one region. Expanding the sample size and conducting studies at different levels in different regions and hospitals would be helpful to obtain more reliable data. Moreover, using a self‐reported questionnaire format may introduce some subjectivity into the results, limiting the generalisability of the findings. Finally, there is no standard for the devices used in ICUs, which may lead to different alarm fatigue among institutions, so the study needs to be repeated in different ICUs.

### 4.2 Implications and Recommendations for Practice

Alarm fatigue reduces the quality of patient care, nurses' qualifications and the continuity of their effectiveness in providing care services and making clinical decisions. Therefore, it is necessary to find a solution to this problem. Nurses' knowledge, attitudes and behaviours regarding clinical alarms are important to combat alarm fatigue. The primary strategy for dealing with alarm fatigue is to reduce the number of alarms. Since the presentation of the IOM report, many studies conducted worldwide have shown that it is not easy to solve alarm fatigue in the triangle of people, technology and organisation. Although no strategic solution has been found for managing alarm fatigue, which still maintains its importance and ranks 4th on the 2024 NPSG list, the research results and suggestions are presented as follows.

− Managers should plan their workloads by considering nurses, providing critical patient care with a staff that will reduce alarm fatigue, preparing an ergonomic and error‐proof work environment and providing rest areas away from noise.
− Nurses should be continuously trained with managerial strategies against frequent alarms, and an institutional culture should be created regarding alarm fatigue.
− Clinicians and medical informatics specialists should establish interdisciplinary teams to develop alarm‐related policies and procedures.

## 5 Conclusion

The study examined the mediating role of role overload in the relationship between medical error tendency and alarm fatigue, which are included in the national patient target list in ICUs. Based on the research findings, it was concluded that the increase in the level of alarm fatigue in nurses caused an increase in role overload, and the increase in role overload caused an increase in medical errors. Our study's results show intensive care nurses' working conditions may affect patient safety. This finding is important considering the intensive care environment's complexity and patient safety's importance. Medical errors are a multifaceted administrative problem that causes the consumption of resources and decreased trust in the health system. Identifying errors in health services and preventing harm is possible by reporting medical errors and analysing work environments. Although intensive care nurses, a profession that can challenge emotional resilience, play a statistically significant role in preventing medical errors, due to the difficulties in working conditions and environments, awareness of potential risks to patients and eliminate these dangers with administrative and organisational interventions during healthcare delivery. The results obtained in our study are consistent with existing studies indicating that nurses' working conditions may increase the risk of medical errors. At the same time, it will help develop effective strategies by better understanding the factors that may lead to medical error tendencies of intensive care nurses responsible for critical patient care.

## Ethics Statement

In order to implement the study, the necessary permissions were obtained from the developers of the three scales used in the data collection tool. In the province where the study would be conducted, written permission was obtained from Ordu University Clinical Research Ethics Committee with the date of 29.02.2024, session number 03 and decision number 2024–26, and from the Public Hospitals affiliated to the Provincial Health Directorate after the approval of the ethics committee. The data were collected by informing the nurses participating in the study that their personal information would be protected and that they could withdraw from the study at any time, and the collection process was carried out without interrupting the functioning of the work environment.

## Conflicts of Interest

The authors declare no conflicts of interest.

## Data Availability

The data that support the findings of this study are available from the corresponding author upon reasonable request.
